# Voluntary exercise fails to prevent metabolic dysfunction‐associated steatotic liver disease progression in male rats fed a high‐fat high‐cholesterol diet

**DOI:** 10.14814/phy2.15993

**Published:** 2024-04-16

**Authors:** Clément Besqueut‐Rougerie, Vivien Chavanelle, Arnaud Michaux, Yolanda F. Otero, Pascal Sirvent, James A. King, Gaël Ennequin

**Affiliations:** ^1^ Valbiotis Riom R&D Center Riom France; ^2^ School of Sport, Exercise and Health Sciences Loughborough University Leicestershire UK; ^3^ Laboratory of the Metabolic Adaptations to Exercise Under Physiological and Pathological Conditions (AME2P), CRNH Auvergne, Chaire Santé en Mouvement Clermont Auvergne University Clermont‐Ferrand France

**Keywords:** inflammation, liver, metabolism, physical activity, steatosis

## Abstract

Metabolic dysfunction‐associated steatotic liver disease (MASLD) is a major public health issue with a worldwide prevalence of 30%–32%. In animal models, voluntary exercise may be an alternative to forced physical activity, avoiding stress, potential injuries, and being logistically simpler. Here, we assessed voluntary exercise (Vex) in Sprague–Dawley rats fed a high‐fat, high‐cholesterol diet for 18 weeks to induce MASLD. We quantified workload (speed and distance) using exercise wheels and evaluated energy expenditure using calorimetric cages. MASLD progression was assessed using circulating and hepatic biochemical and gene markers of steatosis, inflammation, and fibrosis. The animals ran an average of 301 km during the study period, with the average daily distance peaking at 4937 m/day during Weeks 3–4 before decreasing to 757 m/day by the end of the study. Rats exposed to Vex showed no improvement in any of the MASLD‐associated features, such as steatosis, inflammation, or fibrosis. Rats exposed to Vex exhibited a higher total energy expenditure during the night phase (+0.35 kcal/h; *p* = 0.003) without resulting in any effect on body composition. We conclude that, in our experimental conditions, Vex failed to prevent MASLD progression in male Sprague–Dawley rats exposed to a high‐fat high‐cholesterol diet for 18 weeks.

## 1 INTRODUCTION

Metabolic dysfunction‐associated steatotic liver disease, (MASLD) (Rinella et al., 2023), formerly known as nonalcoholic fatty liver disease, NAFLD, has become a major public health issue, with a global prevalence of 30%–32%, yet forecasted to increase as the obesity pandemic expands (Riazi et al., 2022; Younossi et al., 2023). The disease encompasses several stages starting with simple steatotic liver disease (SLD) evolving to metabolic dysfunction‐associated steatohepatitis (MASH, formerly known as nonalcoholic steatohepatitis, NASH). MASH is characterized by the development of lobular inflammation, hepatocyte ballooning, and multiple degrees of fibrosis that result from steatosis, eventually worsening to life‐threatening stages of cirrhosis and liver decompensation (Allen et al., 2022). Altogether, these complications make MASLD the second cause of liver transplant in the US (Doycheva et al., 2018; Younossi et al., 2021). To date, no pharmaceutical treatment has been approved, and only lifestyle modifications are recommended by major health organizations. Among them, physical activity holds a central place, as 150–200 min of moderate‐intensity exercise a week are advised for patients at risk of MASLD (European Association for the Study of the Liver (EASL), European Association for the Study of Diabetes (EASD), European Association for the Study of Obesity (EASO), 2016; Chalasani et al., 2018; Glen et al., 2016), in line with the results of a recent meta‐analysis showing that aerobic exercise improves body weight, liver fat and liver enzymes, in humans with MASLD (Houttu et al., 2022). To investigate new potential therapeutic targets, develop novel drugs, and tailor lifestyle‐based interventions, preclinical models are essential. Human MASLD has successfully been mimicked in rats using high‐fat high‐cholesterol diets (Carreres et al., 2021), also called western‐diet (WD). The authors observed the development of liver steatosis followed by inflammation, ballooning, and fibrosis. In such models, forced physical activity, (motorized treadmill being the most commonly used method) has demonstrated benefits in combating various diseases, including obesity‐related impairments, cancer, and cardiovascular pathologies (Wang et al., 2020). Additionally, a recent systematic review has shown beneficial effects of various forms of forced exercise on liver steatosis, inflammation and fibrosis in several animal models of MASLD (Barrón‐Cabrera et al., 2023). However, forced physical activity protocols display several disadvantages, as they are logistically difficult and time‐consuming to implement, may require prior exclusion of non‐runners, potentially generating a selection bias, and finally, may generate stress and injuries in the process of stimulating animals to keep running, as common methods generally use electric shocks and/or mild hits with a wooden stick. In this context, voluntary exercise (Vex), in the form a free‐access exercise wheel placed in the habitat of the animals, could represent a suitable alternative to forced physical activity, as it allows them to freely exercise by their own will. While voluntary exercise presents the limitation of lacking control over exercise duration and intensity, it allows for the retrospective classification of animals based on their activity levels. This enables the examination of correlations between different exercise components and the resulting metabolic adaptations, thereby empowering researchers to formulate mechanistic hypotheses. In this work, we sought to determine the influence of voluntary running on pathophysiological features of MASLD in Sprague–Dawley rats exposed to a WD diet over 18 weeks. It was hypothesized that Vex would improve MASLD‐associated liver impairments (steatosis, inflammation, and fibrosis). We also provide quantification of biweekly distances and speeds reached by the animals across study period.

## 2 METHODS

### 2.1 Animals and diet

All animal procedures were approved by the local ethical committee under number: #32212 (C2E2A, Auvergne, France). Thirty 8‐week‐old male Sprague–Dawley rats (RjHan: SD) were housed in individual cages and familiarized with the exercise wheel for 2 to 3 days during the first week, before being randomized into three groups: normal diet (ND, *n* = 6: 10% kcal from fat, energy value: 3.8 kcal/g, D09100304, Research Diets, New‐Brunswick, NJ, USA), western‐diet (WD, *n* = 12: 60% kcal from fat, 2% cholesterol, energy value: 5.1 kcal/g; D21021804, Research Diets, New‐Brunswick, NJ, USA) and WD + voluntary exercise (WD‐Vex, *n* = 12, same diet as WD), composition of diets is provided in Table 1. Animals were given ND during the first week of habituation. Number of animals per group was determined based on previous studies having studied the metabolic effect of physical activity on MASLD features in animal models, generally ranging from 10 to 14 (Barrón‐Cabrera et al., 2023), and was reduced to six in group ND to minimize the total number of animals used, in accordance with the 3Rs (Russell & Burch, 1959), because comparisons versus ND do not constitute an objective of this work. Food and water were provided ad libitum for 18 weeks. Rats were checked daily for morbidity/mortality and adverse clinical signs.

**TABLE 1:** Composition of diets.

	ND (D09100304)	WD (D21021804)
Macronutrient	% kcal	% kcal
Protein	20	20
Carbohydrate	70	20
Fat	10	60
kcal/g	3.8	5.1
Ingredient	g	g
Casein	200.00	200.00
L‐Cystine	3.00	3.00
Corn starch	350.00	0.00
Maltodextrin 10	85.00	100.00
Dextrose	169.00	0.00
Sucrose	96.00	93.50
Cellulose	50.00	50.00
Soybean oil	25.00	25.00
Palm oil	0.00	225.00
Lard	20.00	20.00
Mineral mix S10026	10.00	10.00
Dicalcium phosphate	13.00	13.00
Calcium carbonate	5.50	5.50
Potassium citrate	16.50	16.50
Vitamin mix V10001	10.00	10.00
Choline bitartrate	2.00	2.00
Cholesterol	0.00	18.00
Dye	0.05	0.00
Total	1055.05	791.5

### 2.2 Voluntary exercise

Rats belonging to group WD + Vex had free access to a large scurry wheel (80859LS, Lafayette instruments, Lafayette, IN, USA) connected to an electronic sensor and a computer. The number of wheel revolutions per 15 s was recorded constantly for the whole duration of the study with dedicated software (Scurry Activity System, Lafayette Instrument, Lafayette, IN, USA) and data were averaged over 2 weeks. Running distance and speed were calculated every 15 s using wheel circumference (1.44 m). Speed data were then stratified into four intervals:

[0–11.52] m/min, corresponding to [0–2] or (0 ≥ *x* ≥ 2) wheel revolutions per 15 s.

[11.52–17.28] m/min, corresponding to [2–3] or (2 > *x* ≥ 3) wheel revolutions per 15 s.

[17.28–23.04] m/min, corresponding to [3–4] or (3 > *x* ≥ 4) wheel revolutions per 15 s.

>23.04 m/min, corresponding to all values strictly superior to 4 wheel revolutions per 15 s.

Speed intervals were chosen to match running speeds generally used in forced physical activity on a treadmill with no slope. Our first interval, with speeds equal to or lower than 11.52 m/min, was considered low intensity. Moderate‐intensity treadmill protocols commonly range from 14 m/min (Hamada et al., 2020) to 18 m/min (Semeraro et al., 2022) in the literature, corresponding to our second and third intervals ([11.52–17.28] and [17.28–23.04] m/min, respectively). Finally, high‐intensity training protocols are characterized by speeds reaching up to 26 m/min (Verboven et al., 2019), pertaining to our fourth interval (>23.04 m/min).

### 2.3 Body parameters and food intake

Body weight was recorded once weekly. Body composition (fat mass and lean mass) was determined by MRI (Echo MRI, Zynsser Analytic, Fürstenfeldbruck, Germany) every 6 weeks.

Food intake was measured thrice weekly. Daily food intake was calculated by subtracting the leftover amount of food after 2 to 3 days to the weight of distributed diet, divided by the number of days. Energy intake was calculated by multiplying daily food intake by diet energy content (kcal/g). Food efficiency was determined as follows: Food efficiency (g/kcal) = (Total body weight gain (in g))/(Total energy intake (in kcal)).

### 2.4 Indirect calorimetry and locomotor activity

Animals were placed in calorimetric cages (8‐channel multiplex system provided by PromethION, Sable Systems, Las Vegas, NV, USA) for 48 h over Weeks 15–17 for respiratory measurements and estimation of locomotor activity in absence voluntary wheel exercise. Animals belonging to group WD‐Vex had no access to the voluntary exercise wheel during this period. Total energy expenditure (TEE) was calculated using Weir's equation (1949) out of VO_2_ and VCO_2_ measurements for each cage sampled every 30 s. RER was calculated as the ratio of the volume of carbon dioxide (CO_2_) produced to the volume of oxygen (O_2_) used, or VCO_2_/VO_2_. Locomotor activity was estimated with an XYZ beam break array (Sable Systems, Las Vegas, NV, USA). A standard 12‐h light/dark cycle was maintained throughout the calorimetry study.

### 2.5 Fasting glycemia, triglycerides, and total cholesterol

Every 6 weeks, animals were fasted for 5–6 h. Awake rats were restrained in the hand of the experimenter and maintained in a dorsoventral position, while the lower lip was drawn back to expose the gingival vein. The latter was punctured with a 23G needle and whole blood was collected with a capillary tube into an untreated Eppendorf tube. Whole blood was left to clot at room temperature for 30 min before being centrifuged for 10 min at 2000 ***g***. Serum (supernatant) was collected and stored at −80°C until analysis.

#### 2.5.1 Fasting glycemia

Fasting glycemia was assessed using a StatStrip® Glucose Xpress™ (Nova Biomedical Corp., Waltham, USA) from a fresh drop of whole blood from the gum.

#### 2.5.2 Total cholesterol (TC) and triglycerides (TG)

Total serum cholesterol levels were assessed using an enzymatic assay kit provided by Biolabo (#LP80106, Maizy, France). Serum samples were diluted ten times in NaCl 0.9% and absorbance was read in a multiplate reader (Biotek Agilent, Santa Clara, CA, USA). Serum TG levels were assessed using TG enzymatic assay kit provided by Cayman Chem (#10010303, Ann Arbor, MI, USA). Serum samples were diluted ten times in sodium phosphate assay buffer absorbance was read at 540 nm in a multiplate reader (Biotek Agilent, Santa Clara, CA, USA). Duplicate samples that did not fall within 20% of their coefficient of variation were removed.

### 2.6 Euthanasia and tissue sampling

After 18 weeks, animals were fasted for 5–6 h in their habitual cage with access to the exercise wheel for group WD‐Vex. Rats were anesthetized with isoflurane and blood was collected by cardiac puncture. Rats were then euthanized by cervical dislocation and tissues were carefully dissected and snap frozen into liquid nitrogen.

### 2.7 Hepatic parameter analysis

#### 2.7.1 Liver TG and TC

Livers were homogenized in 1 mL of a solution containing 5% NP40 in water. Liver TG levels were determined using an enzymatic assay kit provided by Cayman Chem (#10010303, Ann Arbor, MI, USA), as described. Liver TC levels were assessed using an enzymatic assay Kit provided by Biolabo (#LP80106, Maizy, France) as described.

#### 2.7.2 Liver hydroxyproline

One hundred milligrams of liver was dissolved into 1 mL HCL 6 M for 20 h at 95°C. Lysate was centrifuged for 10 min at 13000 ***g*** at room temperature and supernatant was collected and diluted 2:3 in ultrapure water. Liver hydroxyproline levels were assessed using a Sensitive Tissue Hydroxyproline Assay (#QZBtishyp1, Quickzyme, Leiden, the Netherlands). Absorbance was read at 570 nm in a multiplate reader (Biotek Agilent, USA). Duplicate samples that did not fall within 20% of their coefficient of variation were removed.

#### 2.7.3 Real‐time quantitative PCR

Total mRNA was extracted from livers using TRIzol® (#15596026, Invitrogen, Life Technologies). cDNA was synthesized from 2 μg RNA with the High‐Capacity cDNA transcription kit (#4368814, Applied Biosystems, Life Technologies). PCR amplification was conducted using a CFX system (Bio‐Rad, Hercules, CA, USA) with TaqMan probes (Applied Biosystems, Waltham, MA, USA) and the ΔΔCt method was used to quantify mRNA levels. Gene expression was normalized using *B2m* as a housekeeping gene. Data are represented using the Rq which is normalized to the control group or Rq = 2^−∆∆Ct^ [ΔCt = Ct (target)‐Ct (endogenous); ΔΔCt = ΔCt (sample)‐ΔCt (control)]. TaqMan gene expression assays used in this work are presented in Table 2.

**TABLE 2:** TaqMan gene expression assay references.

Target gene	TaqMan assay reference
*B2m*	Rn00560865_m1
*Ccl2*	Rn00580555_m1
*Il1b*	Rn00580432_m1
*Il6*	Rn01410330_m1
*Lcn2*	Rn00590612_m1
*Tnf*	Rn01525859_g1
*Acta2*	Rn01759928_g1
*Col1a1*	Rn01463848_m1
*Col3a1*	Rn01437681_m1
*Col4a5*	Rn01528524_m1

#### 2.7.4 Histological imaging

Tissues were fixed for 48 h at 4°C in paraformaldehyde and kept in ethanol 70% for 2–3 h before automated tissue processing. They were then embedded in paraffin and sections were cut at 4 μm with a microtome before being stained with hematoxylin and eosin (H&E).

### 2.8 Statistical analysis

Values are presented as mean ± SD. Differences were considered statistically significant at *p* < 0.05. Statistical analysis was performed using GraphPad Prism V.10.0.0. For comparisons of means between groups: Shapiro–Wilk normality test was used to determine whether the data are consistent with a Gaussian distribution. If data were not distributed according to the normal distribution, a Kruskal–Wallis nonparametric test was used followed by Dunn's test for post hoc comparisons. When normal distribution was assumed, measures were subjected to a one‐way ANOVA or Welch‐corrected one‐way ANOVA (if conditions of equivalence of variances were not met) followed by Šídák's or Dunnett's test for multiple comparisons, respectively, when ANOVA reached significance threshold. In case of group comparisons with a categorial variable, two‐way ANOVA, or repeated‐measures two‐way ANOVA was performed followed by Šídák's post hoc test for multiple comparisons. If a sample was missing, making it impossible to run a two‐way ANOVA, a mixed‐effects model followed by Dunnett's test for multiple comparisons was used instead. For all tests, only the following pair‐wise comparisons were run: ND versus WD and WD versus WD + Vex. Group comparison of EE assessed in calorimetric cages was performed by ANCOVA with body weight as covariant, using the online tool provided by the NIDDK Mouse Metabolic Phenotyping Centers (MMPC, www.mmpc.org), using their Energy Expenditure Analysis page (http://www.mmpc.org/shared/regression.aspx) and supported by grants DK076169 and DK115255.

## 3 RESULTS

### 3.1 Voluntary physical activity did not alter body composition and 18‐week food efficiency

**FIGURE 1:**
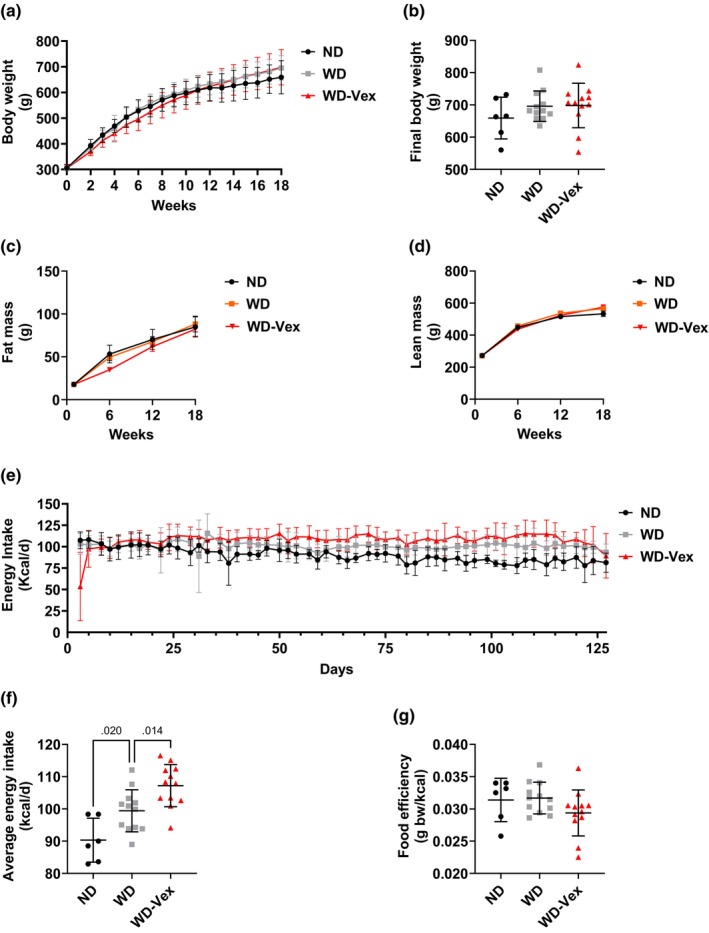
Vex did not alter body composition and 18‐week food efficiency. (a) Body weight measured weekly across study period. (b) Body weight at the end of the study (Week 18). (c) Fat mass and (d) lean mass time courses obtained by echo‐MRI. (e) Energy intake across study period. (f) Average energy intake over study period. (g) Overall food efficiency. One‐way or two‐way ANOVA followed by Šídák's test for comparisons of ND versus WD and WD‐Vex versus WD. Post hoc comparisons were run only whenever ANOVA was significant (*p* < 0.05). BW, body weight; ND, normal diet; WD, western diet; WD‐Vex, western diet and voluntary exercise.

Body weight was recorded weekly over 18 weeks and is presented in Figure 1a. No significant differences were observed between groups at the end of the study (Figure 1b). Body composition was analyzed every 6 weeks by echo‐MRI. Repeated measures two‐way ANOVA interaction was not significant for fat mass (*p* = 0.408), indicating no statistical differences between groups (Figure 1c). Lean mass change over time revealed significant repeated measures two‐way ANOVA interaction (*p* = 0.012), but we did not observe any difference in pairwise post hoc analyses between groups (Figure 1d). Finally, we observed a decrease in energy intake in half (6) of the animals belonging to group Vex in the first days of study (Figure 1e), which may indicate that familiarization with the food hoppers present in exercise wheel cages has not been long enough. All animals displayed normal food intake from Day 8 onward. Average energy intake over study period was significantly higher in WD vs. ND (99.4 ± 6.5 vs. 90.3 ± 6.8 kcal/day, *p* = 0.020) and in WD‐Vex versus WD (107.2 ± 6.5 vs. 99.4 ± 6.5 kcal/day, *p* = 0.014) (Figure 1f). No significant difference was detected between groups in food efficiency (Figure 1g). Additionally, we averaged body weight gain, energy intake and energy efficiency obtained over the first 8 weeks (Weeks 1–8) and the last 10 weeks of study (Weeks 9–18) and observed that Vex significantly reduced food efficiency, compared to WD, solely during the first 8‐week phase (Figure S1d), an effect explained by reduced body weight gain (Figure S1b) in conditions of similar energy intake (Figure S1c). During the second part of the study, energy intake was higher in WD‐Vex, compared to WD, resulting in higher body weight gain in this group (147.8 ± 39.5 vs. 115.8 ± 27.7 g, *p* = 0.079).

### 3.2 Voluntary physical activity did not affect the weight of liver, muscle, and adipose tissue pads

**FIGURE 2:**
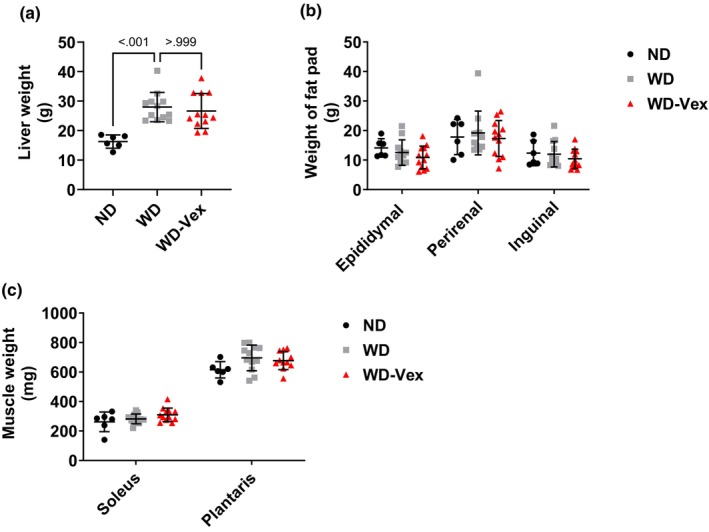
Vex did not affect the weight of liver, muscle, and adipose tissue pads. (a) Liver weight. (b) Epididymal, perirenal, and inguinal fat pad weight. (c) soleus and plantaris weight. Organs were weighed immediately after euthanasia. To determine statistical differences, ANOVA one way followed Šídák's test or Kruskal–Wallis followed by Dunn's test for multiple comparisons. Post hoc comparisons were run only whenever ANOVA or Kruskal–Wallis test was significant (*p* < 0.05). ND, normal diet; WD, western diet; WD‐Vex, western diet and voluntary exercise.

Liver weight was increased by WD exposure, versus ND (27.96 ± 4.96 vs. 16.29 ± 2.25 g, *p* < 0.001); however, no significant improvement was observed with Vex intervention (Figure 2a). Consistent with Echo‐MRI data that showed no changes in fat mass among groups, the weight of epididymal, perirenal and inguinal fat pads were not different among groups (Figure 2b). Similarly, no changes were observed in soleus and plantaris muscles weight (Figure 2c).

### 3.3 Vex quantification

**FIGURE 3:**
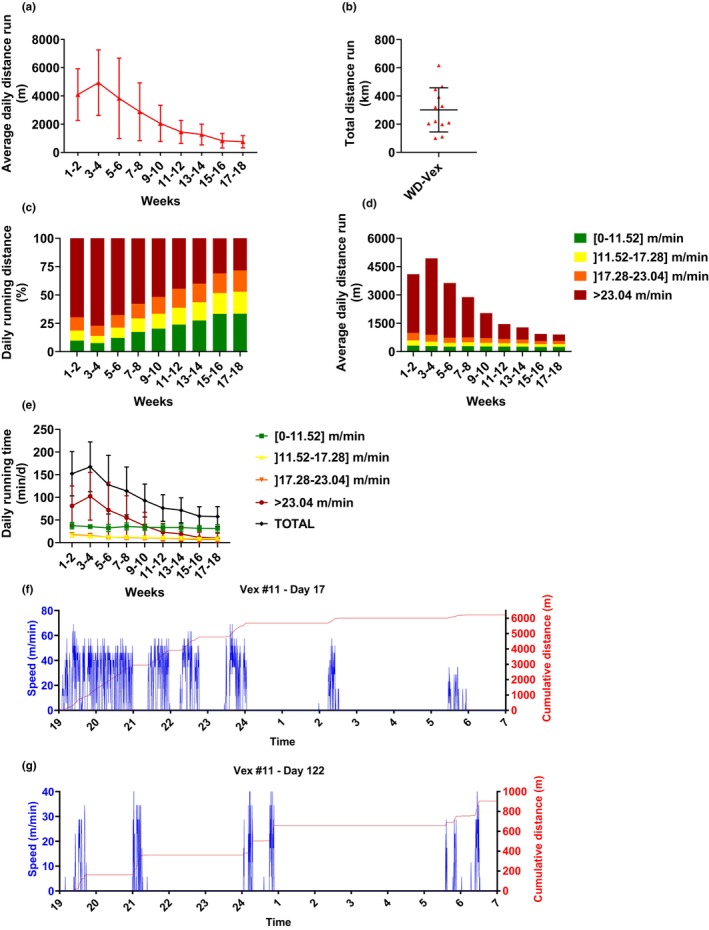
Quantification of Vex. (a) Two‐week average of daily distance run. (b) Total distance run over study period. (c) Percentage of biweekly distance run within different speed intervals. (d) Two‐week average of daily distance run within different speed intervals. (e) Two‐week average daily time spent running within different speed intervals. (f, g) Representative examples of running speeds and cumulative distance achieved over 12 h (19:00–7:00) for WD‐Vex rat #11 during study Day 17 (f, peak activity, Weeks 3–4) and study day 122 (g, Weeks 17–18). No statistical analyses were performed.

In order to characterize workload in WD‐Vex, various parameters related to voluntary running were analyzed and averaged by steps of 2 weeks. Animals reached their maximum activity peak at Weeks 3–4 with a mean distance of 4937 ± 2320 m/day. A large variability among animals was observed (minimum average distance run for Weeks 3–4 was 1498 m/day, maximum was 8502 m/day). Thereafter, the distance gradually decreased to an average of 757 ± 431 m/day at Weeks 17–18 (Figure 3a). At the end of the study, rats had covered a total distance of 301 ± 156 km in average, in 18 weeks (ranging from 101 to 616 km, Figure 3b). Then, we calculated the amount of time and distance run within the different speed intervals: [0–11.52] m/min; [11.52–17.28] m/min; [17.28–23.04] m/min; or >23.04 m/min. During their peak of activity at Weeks 3–4, rats ran an average of 77 ± 14% (4050 ± 2311 m) of their daily distance over >23 m/min. This ratio progressively decreased to 28 ± 20% (341 ± 379 m) at Weeks 17–18 (Figure 3c,d). In line with this, time spent running over 23 m/min decreased from 102 ± 53 min/day during Weeks 3–4 to 10 ± 11 min/day at Weeks 17–18 (Figure 3e). Conversely, the absolute distances (and corresponding time spent) run at low and medium speed intervals ([0–11.52] m/min; [11.52–17.28] m/min; [17.28–23.04] m/min) remained stable all throughout the study (Figure 3e). A representative example of a 12‐h nighttime activity (19:00 to 7:00) on the wheel (15‐s speed and cumulative distance) for WD‐Vex rat #11 is depicted in Figure 3f (peak activity, study Day 17) and Figure 3g (end of study, study Day 122). In this individual, cumulative distance declined from 6218 m (Day 17) to 904 m (Day 122), with marked associated decreases in average and top speed. Additionally, we found that body weight gain was negatively correlated to the total distance run during the 8 first weeks of study (*p* = 0.009, Figure S1e), suggesting an impact of Vex on this parameter, despite individual variability. This correlation was totally abolished during the final 10 weeks of study (*p* = 0.401, Figure S1f).

### 3.4 Vex increased nighttime total energy expenditure (TEE)

**FIGURE 4:**
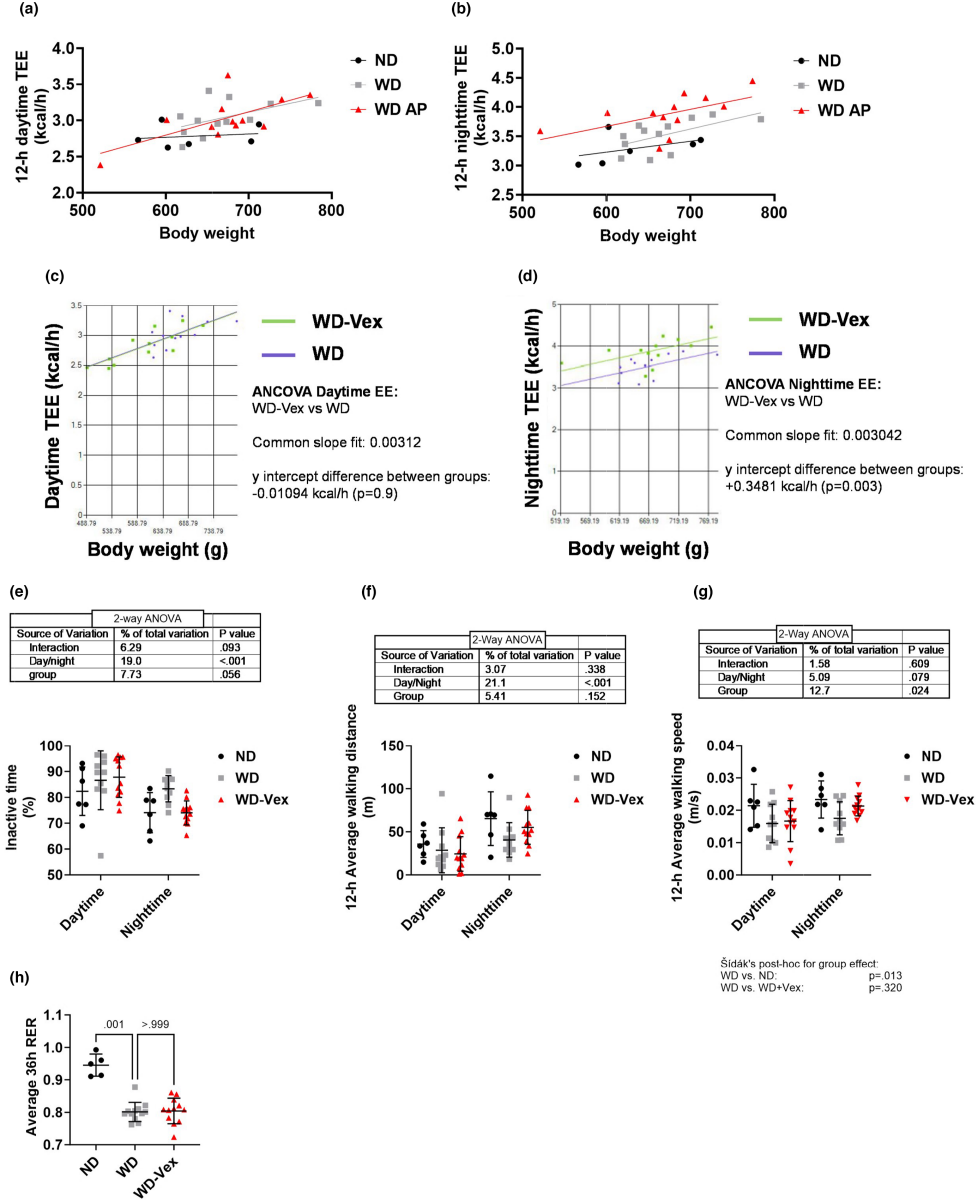
Vex increased nighttime TEE. Rats were placed in calorimetric cages for 48 h (without access to the exercise wheel) for respiratory gas exchanges and locomotion measurements over Week 15–17. (a, b) Average 12‐h daytime (a) and nighttime (b) TEE as a function of body weight. (c, d) TEE of groups WD and WD‐Vex were compared by ANCOVA using body weight as covariant during daytime (c) and nighttime (d) with the online tool provided by the NIDDK Mouse Metabolic Phenotyping Centers (MMPC, www.mmpc.org). Energy expenditure data were analyzed with ANCOVA. (e) Percentage of time spent unmoving in calorimetric. (f) 12‐h average walking distance in calorimetric cages. (g) 12‐h average walking speed in calorimetric cages. Locomotory activity data were analyzed with two‐way ANOVA followed by main group effect Šídák's post hoc test. (h) Average RER during the 36 h of continuous measurement. RER and energy intake were analyzed with Kruskal–Wallis followed by Dunn's multiple comparisons test. ND, normal diet; RER, respiratory exchange ratio; TEE, total energy expenditure; WD, western diet; WD‐Vex, western diet and voluntary exercise.

All rats were housed in calorimetric cages for approximately 48 h in absence of exercise wheel, and data corresponding to a complete 36‐h cycle (12 h night, 12 h day, 12 h night) were analyzed Raw data are presented in Figure 4a (daytime) and Figure 4b (nighttime). WD and WD‐Vex TEE data were analyzed by ANCOVA using body weight as a covariate (Figure 4c,d). We observed no statistical differences in 12‐h daytime TEE (Figure 4c), but ANCOVA revealed a significant increase of TEE in WD‐Vex group compared to WD during night phase (+0.35 kcal/h; *p* = 0.003) (Figure 4b,d). In line with these results, rats belonging to group WD‐Vex tended to spend less time being inactive at night compared to WD (Figure 4e), although this difference did not reach statistical significance threshold (two‐way ANOVA interaction, *p* = 0.093). Conversely, the proportion of inactive time was comparable between WD and WD‐Vex during the light phase, a cycle during which TEE was also similar between both groups. Walking distance was increased at night in all groups (*p* < 0.001, Figure 4f) and walking speed showed a tendency to increase at night as well (*p* = 0.079, Figure 4g) but no significant differences were observed between WD and WD‐Vex for these parameters. Finally, 36‐h average RER was reduced in group WD compared to ND (Figure 4h, 0.80 ± 0.03 vs. 0.95 ± 0.03, *p* = 0.001), while no effect of Vex observed vs. WD (0.80 ± 0.04 vs. 0.80 ± 0.03, *p* > 0.999).

### 3.5 Voluntary exercise did not alter circulating glucose, TG, and TC levels

**FIGURE 5:**
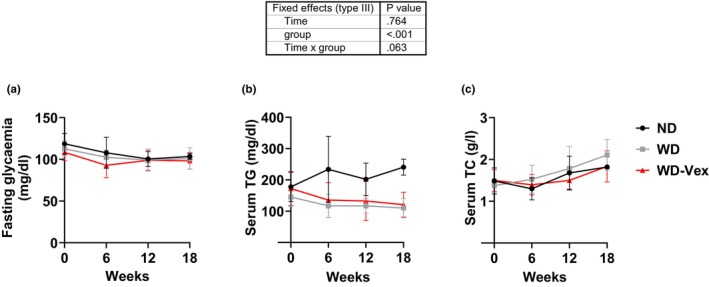
Voluntary exercise did not alter circulating glucose, TG, and TC levels. Animals were fasted for 5–6 h before blood was sampled out. (a) Fasting glycemia. (b) Serum TG levels. (c) Serum TC levels. Mixed‐effects model. ND, normal diet; TC, total cholesterol; TG, triglycerides; WD, western diet; WD‐Vex, western diet and voluntary exercise.

WD feeding for 18 weeks did not induce fasting hyperglycemia in rats, and in this context, no effect of Vex was observed (Figure 5a). Surprisingly, serum TG showed a tendency to be lower in WD compared to ND from Week 6 to the end of the study, although this difference did not reach statistical significance (mixed‐effects model interaction, *p* = 0.063). Here again, no effect of Vex was observed versus WD at any time (Figure 5b). Finally, no significant differences were observed among the groups regarding serum TC level (Figure 5c).

### 3.6 Voluntary exercise did not improve MASLD‐associated hepatic impairments

**FIGURE 6:**
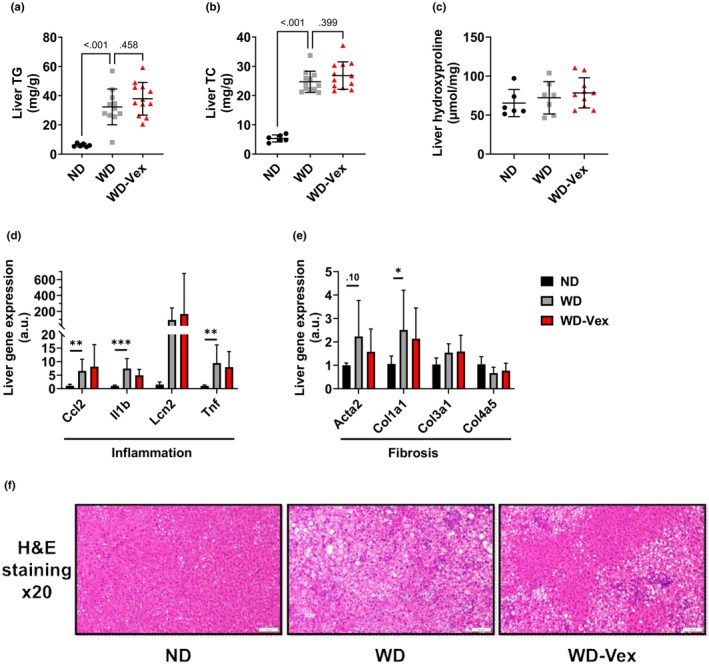
Voluntary exercise did not improve MASLD‐associated hepatic impairments. (a) Hepatic TG. (b) Hepatic TC. (c) Hepatic hydroxyproline. (d) Liver inflammation gene expression assessed by qPCR. (e) Liver fibrosis gene expression assessed by qPCR. (f) Representative images of H&E‐stained liver sections (×20 magnification). One‐way ANOVA or Welch‐corrected one‐way ANOVA or Kruskal–Wallis followed by Šídák's or Dunnett's or Dunn's test for multiple comparisons, respectively. ND versus WD: *p < 0.05, **p < 0.01, ***p < 0.001. A.u., arbitrary units; H&E, hematoxylin & eosin; ND, normal diet; TC, total cholesterol; TG, triglycerides; WD, western diet; WD‐Vex, western diet and voluntary exercise.

Liver TG and TC were increased in group WD, versus ND (32.3 ± 12.2 vs. 6.10 ± 1.10 mg/g of liver, *p* < 0.001, and 24.7 ± 3.63 vs. 5.35 ± 1.19 mg/g of liver, *p* < 0.001, respectively), but no improvements were observed in group WD‐Vex (Figure 6a,b). Liver hydroxyproline, a major component of fibrillar collagen, did not show any difference among the groups (Figure 6c). Representative images of H&E‐stained liver sections are provided in Figure 6f. We also assessed the expression of different inflammation and fibrosis gene makers in the liver and found elevated *Ccl2*, *Il1b*, and *Tnf* in group WD vs. ND (*p* = 0.004, *p* < 0.001 and *p* = 0.004, respectively, Figure 6d). Similarly, we found increased expression of *Col1a1* in WD versus ND (*p* = 0.022, Figure 6e) and *Acta2* gene expression tended to increase in WD, compared to ND (*p* = 0.10, Figure 6e). However, no effect of Vex was observed on any of these markers.

## 4 DISCUSSION

In recent years, lifestyle modifications and particularly physical activity have emerged as the most viable way of treating MASLD, given that no approved pharmaceutical treatments are available for patients, to date. However, our hypothesis that voluntary exercise would exert beneficial effects on diet‐induced MASLD in Sprague–Dawley rats was not supported by the results of the study.

Rats consuming WD for 18 weeks displayed liver steatosis, elevated expression of inflammation and fibrosis gene markers, but implementing Vex did not result in any improvement. We did observe an increase in average energy intake in the group WD‐Vex compared to WD, which did not translate into higher body weight or fat mass. We postulate that the energy intake difference may therefore correspond to the increased TEE associated with physical activity, as previously described (Swallow et al., 2001). In addition, gas exchange analysis in calorimetric chambers (without access to the exercise wheel) showed that TEE was increased in WD‐Vex at night only. Interestingly, this result could be associated with a trend towards a reduction of the amount of inactive time in this group (not reaching statistical significance), during the night phase. This could be indicative of a higher activity level during the night phase for rats exposed to Vex despite the absence of a voluntary exercise wheel, or a potential stress component due to the sudden deprivation of the exercise wheel, an environmental enrichment. In any case, these increases in REE and activity did not result in any improvements in body composition, circulating parameters, or MASLD hepatic markers, in this model. Noteworthily, we also found no increase in fat mass or detrimental effects on fasting glucose and circulating lipids induced by WD (compared to ND). Counter‐intuitively, and although the difference did not reach statistical significance, serum TG tended to be decreased in WD‐exposed rats, compared to ND, a result also reported by other teams (Fungwe et al., 1993; Wang et al., 2010). While we do not provide here a definitive answer, this intriguing phenomenon could be related to the absence of cholesteryl ester transfer protein (CETP) in rats (Briand et al., 2018; Haa & Barter, 1982), a protein that regulates circulating lipid profile by exchanging triglycerides from very‐low‐density cholesterol for cholesteryl esters from high‐density lipoproteins (HDL) particles (Weber et al., 2010). We speculate that the lack of this protein could participate in the abnormal serum TG response following WD exposure. Incidentally, in humans, the use of CETP pharmacological inhibitors results in reduced circulating triglyceride levels (Cannon et al., 2010; Nicholls et al., 2011). In conclusion, MASLD features in this model seem to have developed in absence of many obesity‐related impairments. With this in mind, we cannot rule out that Vex would have elicited beneficial effects in a different model of diet‐induced obesity. Additionally, we cannot rule out the possibility that acute exercise rats in group WD‐Vex did not impact our results. Indeed, animals had continuous access to the exercise wheel including during fasting periods preceding blood draws or euthanasia. The existing literature surrounding the present topic is scarce. To our knowledge, only two teams have studied the effects of Vex in rats with MASLD. One team used CCK1‐receptor Knock‐out hyperphagic rats (Otsuka Long Evans Tokushima Fatty, OLETF) exposed to Vex for 36 weeks (Rector et al., 2011) or 16 weeks (Rector, Thyfault, Morris, et al., 2008). The authors reported several features of human MASLD in OLETF rats and Vex was successful in triggering improvements in glucose tolerance, liver steatosis, ballooning, and fibrosis. This team published several other articles derived from these two experiments, with different foci, such as adipose tissue and Type 2 diabetes (Laye et al., 2009), liver FGF21 signaling (Fletcher et al., 2012), or the effect of exercise cessation (Morris et al., 2008; Rector, Thyfault, Laye, et al., 2008). Another research team used a liquid high‐fat diet to induce MASLD in Sprague–Dawley rats. They compared Vex (17 weeks) to forced treadmill training (8 weeks consecutive to 9 weeks of sedentary lifestyle). The authors concluded that 8 weeks treadmill training was more efficient than 17 weeks Vex in eliciting benefits on MASH features assessed by histological scoring (Gonçalves et al., 2015; Gonçalves, Passos, et al., 2014). Here again, several other publications have emerged from this experiment, exploring various topics (Gonçalves et al., 2016; Gonçalves, Maciel, et al., 2014; Passos et al., 2015), but no further assessment of MASLD hallmarks was provided. Contrary to our present work, those studies did not provide any quantification of Vex other than average weekly distance (not reported in all the above‐mentioned studies). Hence, to our knowledge, this study is the first to have quantified voluntary distance run by speed categories in Sprague–Dawley rats, in a context of diet‐induced MASLD.

Noteworthily, we observed a high variability among animals regarding the distance run in the exercise wheel, especially during their activity peak in the first half of the study. We also noted that the high activity phase (roughly, during the first 8 weeks of study) was characterized by a greater amount of time running at higher speeds (over 23 m/min), while absolute distances run at moderate and lower intensities remained stable over the 18 weeks of study. Aging typically leads to an early decline in performance, including in rats, as demonstrated in 24‐ to 30‐month‐old Brown Norway × Fischer rats by Carter et al (2002). Our data, obtained in 2‐ to 6‐month‐old Sprague–Dawley rats, show a decline in performance noticeable after as early as 8 weeks of training (4‐months of age rats). Other teams have observed such decline in voluntary exercise performance in Sprague–Dawley rats fed normal chow (Masini et al., 2011) or a WD (Gonçalves et al., 2015). To our knowledge, the cause of this decline has not been studied. We could speculate that WD exposure may have accelerated this phenomenon, as suggested by a study in Wistar rats fed a high‐fat diet and forced‐exercised on a treadmill (Murray et al., 2009), a definitive way of addressing this question in our model would be to add an exercising age‐matched group of rats fed a ND. In this context, certain pieces of our data may suggest that a shorter study duration (limited to the first active 8‐week phase, Figure S1a) could have been a better design to observe metabolic adaptations following Vex. Indeed, the decrease in voluntary activity combined with the upregulated energy intake in group WD‐Vex during the second part of the study (Weeks 9–18) may have blunted the beneficial effects obtained during the first part of the experiment. In line with this, body weight gain negatively correlated with total distance run only during the first 8 weeks of study (Figure S1e,f). Together, these results suggest that Vex may have elicited dose‐dependent effects on food efficiency and body weight within the initial 8 weeks of study, and that those eventually dissipated as the activity level dropped during the last 10‐week phase. The beneficial effects of voluntary exercise on body weight over the first part of the study are likely due to increased physical activity‐driven energy expenditure but could also be related to changes in intestinal macronutrient absorption. Unfortunately, none of these parameters were assessed in this work, thereby limiting the reach of this explanatory hypothesis.

Exercise intensity comparison with forced treadmill training is delicate, as treadmill sessions are limited to a short period of time per day, typically less than 1 hour, as opposed to the 12‐h nighttime active phase in our study. They involve slower speeds, which sometimes makes experimenters opt for a positive or negative incline to tailor workload and the type of muscle contraction (Chavanelle et al., 2014). With this in mind, it remains nonetheless clear that, contrary to Vex in our study, a large number of treadmill training protocols in various rat models of MASLD have shown positive results on hepatic or circulatory markers of the pathology (Kalaki‐Jouybari et al., 2020; Linden et al., 2015, 2016; Nikroo et al., 2020), and, as mentioned hereabove, other teams using Vex in rat models of MASLD have succeeded in halting progression of the pathology (Abdulqader et al., 2023; Rector et al., 2011). While this work was not designed to elucidate the mechanisms behind the absence of positive effects of Vex in our model, we can speculate, based on our results, that the fact that the rats continued their same level of caloric intake in the second half of the study while their running distance greatly diminished may have offset the potential benefits obtained during the first 8 weeks of study.

In addition, while this study provides new insights into understanding Vex in preclinical models of MASLD, the observation of liver impairments in absence of significant obesity and deterioration of associated features (hyperglycemia, dyslipidemia) represents a major limitation to the scope of our conclusions. This questions the relevance of this model for mimicking human MASLD, intricately related to the development of obesity and metabolic syndrome (Godoy‐Matos et al., 2020). In this regard, this work does not challenge physical activity recommendations in humans, nor does it refute the beneficial effects of different types of exercise obtained in the above‐mentioned previous studies in animal models.

In conclusion, we show in this work that free access to a voluntary exercise wheel failed to improve MASLD‐associated features in male Sprague–Dawley rats exposed to a high‐fat high‐cholesterol diet for 18 weeks. Our characterization of voluntary exercise in rats includes detailed data on the distances covered and time spent running within different speed intervals. Our results may indicate a progressive decline in the proportion of higher‐intensity exercise possibly associated with aging, exposure to a WD, or the combination of both. We hope that this approach can be used in future preclinical research to allow better comparison of exercise intensities across different training protocols.

## FUNDING INFORMATION

This study was funded by the French National Research Agency (ANR‐within the call for projects “France 2030–Plan de relance–mesure de preservation de l'emploi R&D”) and the Valbiotis Society.

## CONFLICT OF INTEREST STATEMENT

VC, AM, YFO and PS are Valbiotis employees. CBR was completing a six‐month internship at Valbiotis.

## ETHICS STATEMENT

All animal procedures were approved by the local ethical committee under number: #32212 (C2E2A, Auvergne, France).

## Supporting information


Figure S1.

## Data Availability

The data that support the findings of this study are available from the corresponding author upon reasonable request.
