# Supplementary material for: Voluntary exercise fails to prevent metabolic dysfunction‐associated steatotic liver disease progression in male rats fed a high‐fat high‐cholesterol diet
Source: Physiol Rep. 2024 Apr 16;12(8):e15993. doi: 10.14814/phy2.15993 (PMC11021195; doi:10.14814/phy2.15993)
Supplement: Supplementary file 1 — Figure S1. [file PHY2-12-e15993-s001.pdf]

Supplementary data

Supplementary figure

**Figure S1. Comparison of high and low activity phases.** A: High (week 1 to week 8) and low (week 9 to week 18) activity phases were determined based on running distance and speed. B: Body weight gain during high activity phase (week 1 to 8) vs. low activity phase (week 9 to 18). C: Energy intake during high activity phase (week 1 to 8) vs. low activity phase (week 9 to 18). D: Food efficiency during activity phase (week 1 to 8) vs. low activity phase (week 9 to 18). Two-way ANOVA followed by Šídák's post-hoc test. E: Linear regression analysis of body weight gain during week 1 to 8 and total distance run during the same period. F: Linear regression analysis of body weight gain during week 9 to 18 and total distance run during the same period. ND: normal diet; WD: western diet; WD-Vex: western diet and voluntary exercise.

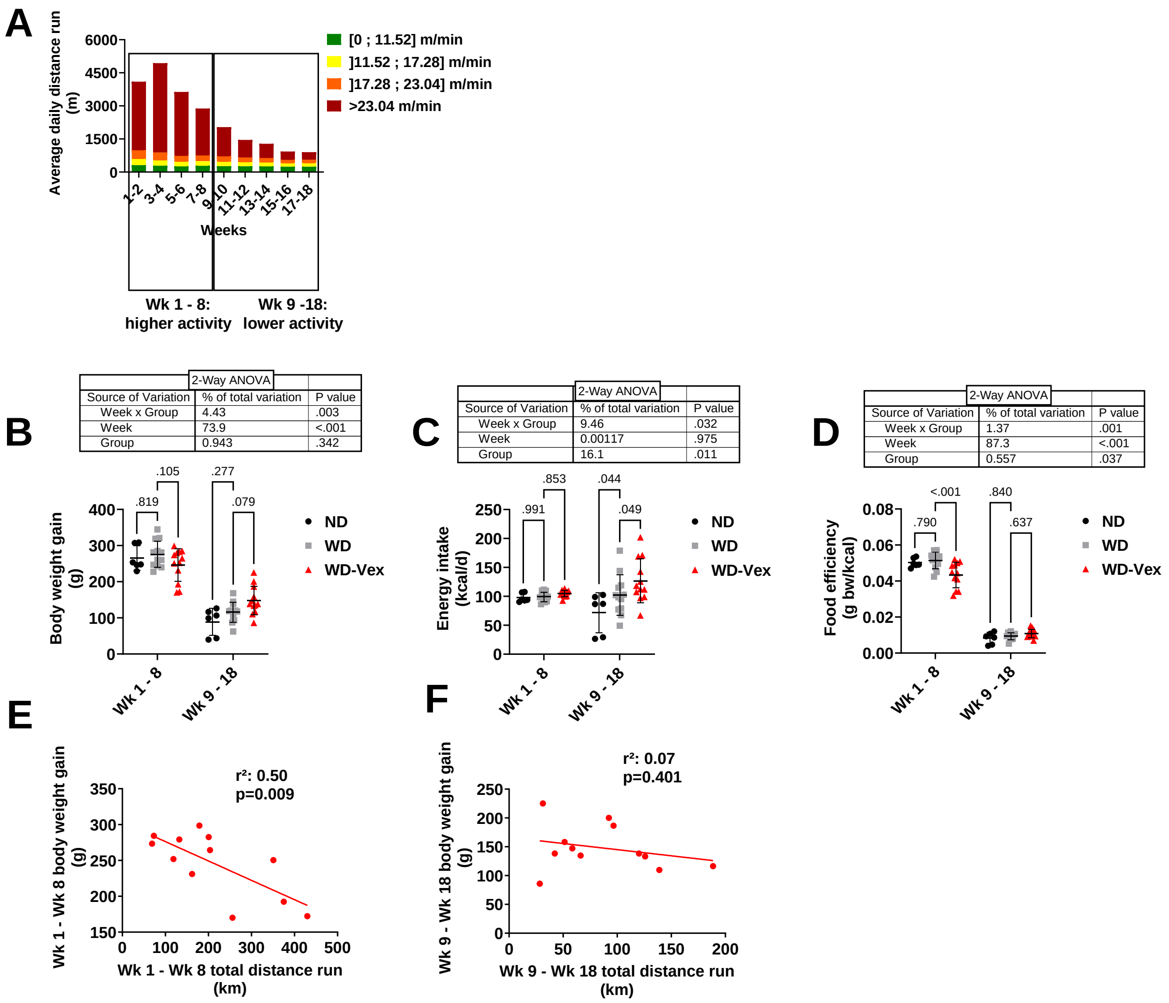


B — 2-Way ANOVA

| Source of Variation | % of total variation | P value |
|---|---|---|
| Week x Group | 4.43 | .003 |
| Week | 73.9 | <.001 |
| Group | 0.943 | .342 |

C — 2-Way ANOVA

| Source of Variation | % of total variation | P value |
|---|---|---|
| Week x Group | 9.46 | .032 |
| Week | 0.00117 | .975 |
| Group | 16.1 | .011 |

D — 2-Way ANOVA

| Source of Variation | % of total variation | P value |
|---|---|---|
| Week x Group | 1.37 | .001 |
| Week | 87.3 | <.001 |
| Group | 0.557 | .037 |
